# Editorial: Germline Development: From Germline Stem Cells to Gametes

**DOI:** 10.3389/fcell.2020.00650

**Published:** 2020-08-06

**Authors:** Myon-Hee Lee, Rosa E. Navarro, Sung Min Han

**Affiliations:** ^1^Division of Hematology and Oncology, Department of Medicine, Brody School of Medicine at East Carolina University, Greenville, NC, United States; ^2^Departamento de Biología Celular y Desarrollo, Instituto de Fisiología Celular, Universidad Nacional Autónoma de México, Mexico City, Mexico; ^3^Department of Aging and Geriatric Research, College of Medicine at the University of Florida, Gainesville, FL, United States

**Keywords:** germline development, germline stem cell, differentiation, sex determination, gametogenesis

Stem cells are endowed with the unique ability to generate daughter cells and self-renew. In the adult gonads of many organisms, a specialized population of stem cells (termed germline stem cells; GSCs) is maintained to replenish the reservoir of germ cells whose numbers are depleted by gamete production. As parent cells of gametes (oocytes and sperm), GSCs are also responsible for the transgenerational flow of genetic information. These cells make several major fate decisions—the mitosis-meiosis, the sperm-oocyte, and the apoptosis-survival decisions. A strict regulation of these decisions is vital to the development of all multicellular organisms, including humans. Aberrant regulation, on the other hand, results in either loss of a specific cell type or uncontrolled cell proliferation; two events that are associated with infertility and germline tumors, respectively. Although significant progress has been made in dissecting the molecular mechanisms of each type of regulation in worms, flies, zebrafish, and mice, understanding the intricate interplay among the regulatory networks during germline development remains a major challenge.

Gonadogenesis is a unique developmental process that gives rise to one of two distinct reproductive tissues, the testis or ovary, from a common bipotential precursor. This complex process begins with the establishment of genetic sex at the time of fertilization. For this purpose, several conserved genes and pathways play complementary roles in the initial decision to develop as a testis or as an ovary, in cross-repression of the testis and ovary developmental pathways, and in the maintenance of gonadal phenotypes after birth (Wilhelm et al., 2013). In this issue, Qiu et al., report that an NPXY motif of β-integrin also contributes to gonad morphology and male-specific tail structure.

During germline development, conserved signaling pathways, including Notch, Wnt, JAK-STAT, and TGFβ, promote mitotic division of germ cells at the expense of meiosis. These extrinsic signals also activate intrinsic regulators in GSCs. One of the best-studied regulators is the PUF (Pumilio/FBF) family of RNA-binding proteins. PUF proteins are required to maintain GSCs in worms and flies (Lin and Spradling, 1997; Crittenden et al., 2002), and their conserved functions have recently been identified in vertebrate stem cells (Lee et al., 2019). In this issue, Wang and Voronina review the multifaceted roles of *C. elegans* PUF proteins in GSCs and progenitor cells, and discuss the factors accounting for their distinct biological functions. Notably, Park et al. demonstrate that PUF-8, in *C. elegans* germline, and its repressing target mRNA, *gld-2*, are required to maintain meiotic cell state and to inhibit the regression to mitotic cells (leading to germline tumors) via dedifferentiation.

Once germ cells commit to meiotic division, their fate (sperm or oocytes) is resolved by sex determination regulators. This mechanism has been well-studied in *C. elegans* due to its hermaphroditism. *C. elegans* hermaphrodites produce both sperm and oocytes in the same gonad, and many of the sex determination regulators are active during the post-transcriptional stage. Furthermore, PUF proteins also promote the oocyte fate by inhibiting the mRNA translation of sperm fate-promoting genes. These observations suggest that post-transcriptional regulation seems to be a major event in *C. elegans* germline development. *Drosophila* germlines also have contributed to gametogenesis. In this issue, Hinnant et al. discuss the molecular mechanism by which cell cycle control is integrated with germ cell polarity and fate to maintain oocyte production.

To ensure successful transfer of genetic information, germ cells have developed elaborate defense mechanisms to counteract adverse growth conditions. In this issue, Carranza-García and Navarro review how *C. elegans* germline responds to starvation. Under conditions of energy depletion, gonads display severe atrophy due to arrest of GSC proliferation and impaired meiosis. Upon nutrient restoration, however, gonads regenerate and animals regain fertility. Moreover, it is also shown that germ cell apoptosis plays an important role during the oogenic starvation response particularly as it preserves oocyte integrity.

Ribonucleoprotein (RNP) granules are broadly conserved, non-membranous organelles formed by phase separation. The formation of these granules contributes to the preservation of gamete integrity. In this issue, Schisa summarizes how worms protect their mRNAs and proteins by forming RNP granules, how such an occurrence regulates gene expression, and also how meiotic arrest, heat shock, and nutrient deprivation influence the formation of RNP granules in male and female germ cells of diverse organisms. In addition, Huggins and Keiper review the role of germ cell eIF4Es and non-canonical forms of its regulatory binding protein 4EBP in translation regulation, and how they influence or are influenced by RNP complex composition.

In summary, this special topic stimulates the continuing efforts to understand the molecular mechanisms of how GSCs and their cell fates are systemically controlled in vertebrates and invertebrates. Some remarkably conserved mechanisms and intriguingly exciting differences are beginning to emerge from studies of germlines in *C. elegans, Drosophila*, zebrafish, and mice. Given that germline development in these model organisms is also broadly conserved with that in humans, animal studies are thus invaluable to decipher the connection between germline development and tumorigenesis.

## Author Contributions

M-HL, RN, and SH wrote and edited the manuscript. All authors contributed to the article and approved the submitted version.

## Conflict of Interest

The authors declare that the research was conducted in the absence of any commercial or financial relationships that could be construed as a potential conflict of interest.
